# Kinlessness at the End of Life in the United States: Implications for Place of Death, and Quality of Life Among Older Adults

**DOI:** 10.1093/geroni/igaa057.2037

**Published:** 2020-12-16

**Authors:** Katherine Ornstein, Natalie Plick, Claire Ankuda

**Affiliations:** Icahn School of Medicine at Mount Sinai, New York, New York, United States

## Abstract

We used the Health and Retirement Study, a large nationally representative study of U.S. older adults from 2002-2015, to identify decedents and assess quality of EOL care by availability of kin. 7.9% of participants were kinless at EOL (no adult children or spouses), reflecting national estimates of 1,027,600 older adults. Those who were kinless at EOL were more likely to be female (61.2% vs 51.5%), from the lowest wealth quartile (53.6% vs 35.6%), and less likely to be white and non-Hispanic (75.6% vs 81.8%). Among the community-dwelling population, individuals with kin received 2.4 times as much hours of help from informal caregivers per month, compared to those without kin. We did not observe differences in rates of hospital death by kin status in adjusted models. More work is needed to assess any unmet needs in the EOL period for kinless older adults, especially as healthcare moves towards increased in-home supports.

